# Autocrine epiregulin activates EGFR pathway for lung metastasis via EMT in salivary adenoid cystic carcinoma

**DOI:** 10.18632/oncotarget.7940

**Published:** 2016-03-06

**Authors:** Shuli Liu, Dongxia Ye, Dongliang Xu, Yueling Liao, Ling Zhang, Liu Liu, Wenwen Yu, Yanan Wang, Yue He, Jingzhou Hu, Wenzheng Guo, Tong Wang, Beibei Sun, Hongyong Song, Huijing Yin, Jingyi Liu, Yadi Wu, Hanguang Zhu, Binhua P. Zhou, Jiong Deng, Zhiyuan Zhang

**Affiliations:** ^1^ Department of Oral and Maxillofacial–Head and Neck Oncology, Ninth People's Hospital, Shanghai Jiao Tong University School of Medicine, Shanghai, China; ^2^ Key Laboratory of Cell Differentiation and Apoptosis of Chinese Minister of Education, Shanghai Jiao Tong University School of Medicine, Shanghai, China; ^3^ Shanghai Key Laboratory for Tumor Microenvironment and Inflammation, Shanghai Jiao Tong University School of Medicine, Shanghai, China; ^4^ Translation Medicine Center, Shanghai Chest Hospital, Shanghai Jiao Tong University, Shanghai, China; ^5^ Department of Molecular and Cellular Biochemistry, Markey Cancer Center, University of Kentucky College of Medicine, Lexington, KY, USA

**Keywords:** SACC, lung metastasis, EREG, EGFR, EMT

## Abstract

Salivary adenoid cystic carcinoma (SACC) is characterized by invasive local growth and a high incidence of lung metastasis. Patients with lung metastasis have a poor prognosis. Treatment of metastatic SACC has been unsuccessful, largely due to a lack of specific targets for the metastatic cells. In this study, we showed that epidermal growth factor receptors (EGFR) were constitutively activated in metastatic lung subtypes of SACC cells, and that this activation was induced by autocrine expression of epiregulin (EREG), a ligand of EGFR. Autocrine EREG expression was increased in metastatic SACC-LM cells compared to that in non-metastatic parental SACC cells. Importantly, EREG-neutralizing antibody, but not normal IgG, blocked the autocrine EREG-induced EGFR phosphorylation and the migration of SACC cells, suggesting that EREG-induced EGFR activation is essential for induction of cell migration and invasion by SACC cells. Moreover, EREG-activated EGFR stabilized Snail and Slug, which promoted EMT and metastatic features in SACC cells. Of note, targeting EGFR with inhibitors significantly suppressed both the motility of SACC cells *in vitro* and lung metastasis *in vivo*. Finally, elevated EREG expression showed a strong correlation with poor prognosis in head and neck cancer. Thus, targeting the EREG-EGFR-Snail/Slug axis represents a novel strategy for the treatment of metastatic SACC even no genetic EGFR mutation.

## INTRODUCTION

Salivary adenoid cystic carcinoma (SACC) is a common subtype of malignant salivary gland tumors in head and neck, accounting for approximately 10% of salivary gland tumors and about 25% of malignant salivary gland tumors [1]. Clinical studies indicate that SACC is characterized by invasive local growth and a high incidence of distant metastasis [2]. Although SACC can metastasize to multiple organs including bone, liver, and cerebrum, the lung is the predominant anatomical site of distant metastasis [3]. Lung metastasis is also a poor prognostic factor for patients with SACC. Patients without distant metastasis have overall 5-, 10-, and 20-year survival rates of 85.6%, 67.4%, and 50.4%, respectively, whereas the survival rates for patients with distant metastasis are 69.1%, 45.7%, and 14.3%, respectively [4]. To date, treatment for lung metastases has been unsuccessful, mainly due to a lack of specific targets for the metastatic cancer cells. Thus, an understanding of the mechanisms that govern lung metastasis in SACC is necessary for development of novel targeting strategies and improve patient survival.

Cancer metastasis consists of at least four distinct steps: invasion, intravasation, extravasation, and metastatic colonization [5, 6]. The first step, the acquisition of invasive capability and motility, is the rate-limiting step in the metastatic cascade [7]. Epithelial-mesenchymal transition (EMT) is a process vital for morphogenesis during embryonic development, and is also critically important for metastasis [8]. A hallmark of EMT is the loss of E-cadherin expression [9]. Several transcription factors are implicated in the transcriptional repression of E-cadherin, including the zinc finger proteins of Snail, Slug, Twist, δEF1/ZEB1, SIP1, and E12/E47 [10–17]. EMT is also a dynamic and reversible process, provoked by signals, such as TGF-β, Wnt and TNF-α, from the microenvironment [18–22]. However, the cellular source of signals that control the EMT cascade in specific cancers are not completely understood.

Aberrant activation of EGFR is involved in tumor development and progression for many cancers. Activation of EGFR can result from genetic mutation and gene amplification. Epiregulin (EREG) functions as a ligand of the EGF receptors EGFR/HER1 and HER4. EREG binds directly to EGFR and HER4 to induce tyrosine phosphorylation (activation) of EGFR, HER2, HER3 and HER4 [23, 24]. EGFR ligands implicated in human cancers, include EGF, EREG, amphiregulin (AREG), heparin-bound EGF (HB-EGF) and transforming growth factor alpha (TGFα) [25]. The EGF ligand family participates in tumor proliferation, migration, invasion and angiogenesis. For example, EGF secreted by endothelial cells can promote cancer metastasis by inducing EMT in head and neck cancers [26]. EREG stimulates the proliferation of fibroblasts, hepatocytes, smooth muscle cells and keratinocytes, but inhibits the growth of several tumor-derived cell lines [27, 28]. Many studies show that EREG expression correlated with cancer metastasis and could serve as a prognostic marker for cancers such as breast, colorectal, bladder and oral cancers [29–31]. However, the cellular source of EREG and the mechanism by which EREG promotes cancer metastasis are still unclear.

We hypothesize that EMT plays an important role in the metastasis of SACC. In this study, we used two homologous SACC cell lines, SACC-83 and SACC-LM, to characterize the metastatic features of SACC [32]. The parental cell line, SACC-83 with a low lung metastasis rate, was isolated from a patient diagnosed pathologically with SACC in the sublingual gland. The daughter cell line SACC-LM, which has a high lung metastatic rate, originated from a murine lung metastatic lesion after a 5 round selection process, starting from intravenous injection of SACC-83 cells [32]. With these two SACC lines, we found that autocrine EREG-induced EGFR activation promotes lung metastasis via EMT. Moreover, upregulated EREG is prevalent in head and neck cancer and correlates with poor survival.

## RESULTS

### Lung metastatic SACC cells exhibit EMT-like characteristics

**Figure 1:**
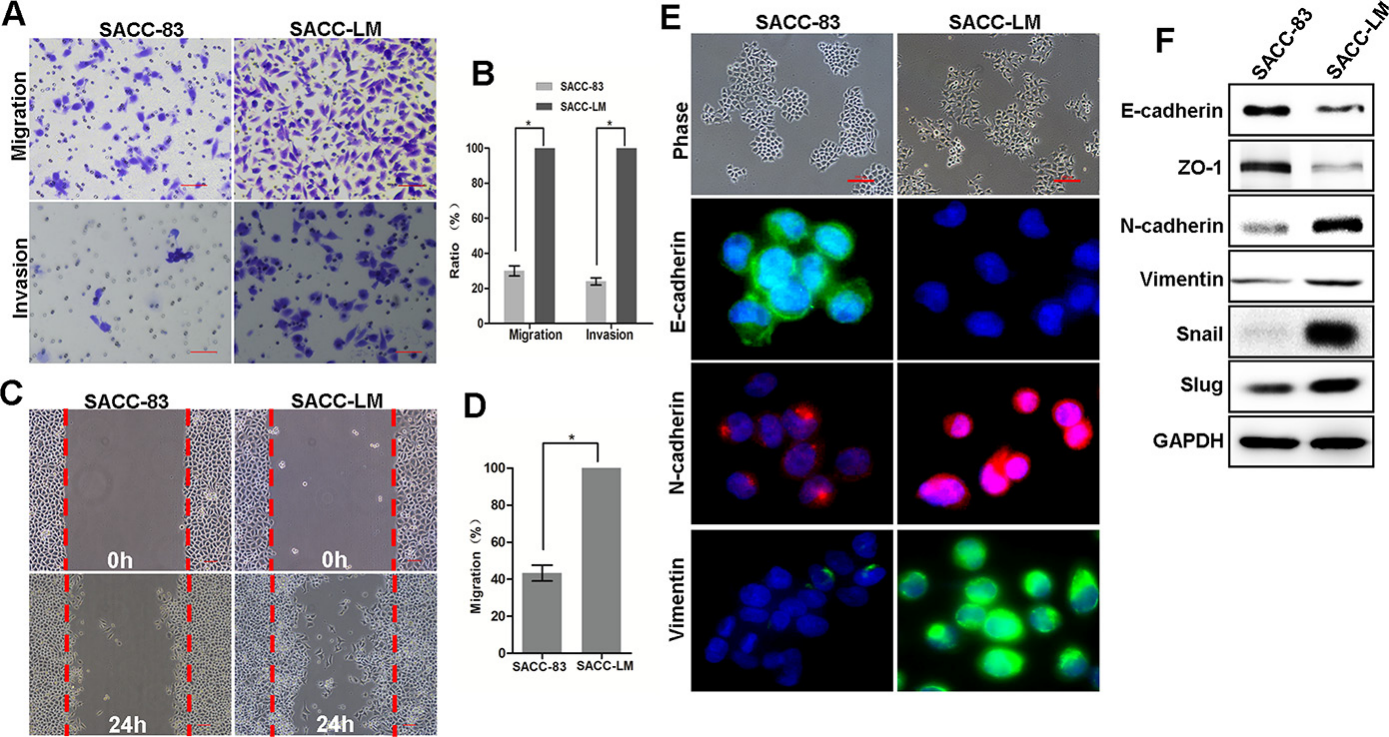
Lung metastatic SACC-LM cells exhibit EMT characteristics (**A**) The transwell migration and invasion assays established the migration and invasion capability of SACC-83 and SACC-LM cells with representative images shown. Scale bar = 200 μm. (**B**) Graphic representation of the percent of migrated cells from 3 separate experiments (mean ± SD). * indicates a *p* < 0.05. (**C**) Representative images of wound healing for SACC-83 and SACC-LM cells. Scale bar = 200 μm. (**D**) The number of migrated cells within the areas of healing surpassing the red lines was determined, and each experiment was repeated 3 times. * indicates a *p* < 0.05. (**E**) Representative images of the morphology and staining for E-cadherin, N-cadherin and vimentin in SACC-83 and SACC-LM cells. Scale bar = 200 μm. (**F**) Western blot analysis of E-cadherin, N-cadherin, ZO-1, vimentin, Snail and Slug protein levels in SACC-83 and SACC-LM cell lines.

To investigate the mechanisms that underlie lung SACC metastasis, we selected SACC-83 and SACC-LM as a pair of cell lines for characterization. First, we examined an EMT-related function, the migration capability of SACC-83 and SACC-LM cells, using the transwell and wound-healing assays. The transwell analysis showed that SACC-LM cells were much more motile and invasive than SACC-83 cells (Figure 1A–1B) *in vitro* and in areas of healing (Figure 1C–1D). In culture, SACC-83 cells exhibited the typical polygonal morphology of epithelial cells (Figure 1E), and immunofluorescence analysis revealed high levels of the epithelial marker E-cadherin and low levels of mesenchymal markers, N-cadherin and vimentin, as indicated. In contrast, SACC-LM cells were scattered, displayed a fibroblast-like morphology, with low levels of E-cadherin and high levels of N-cadherin and vimentin (Figure 1E). Immunoblot analysis confirmed the molecular features of these two cell lines (Figure 1F). Consistently, SACC-LM cells showed increased expression of Snail and Slug and repressed expression of E-cadherin (Figure 1F). Taken together, these data indicate that SACC-LM cells exhibited increased EMT-like characteristics compared to SACC-83 cells. Thus, EMT may be involved in SACC-LM lung metastasis.

### Autocrine EREG activates EGFR pathway in high metastatic SACC-LM cells

**Figure 2:**
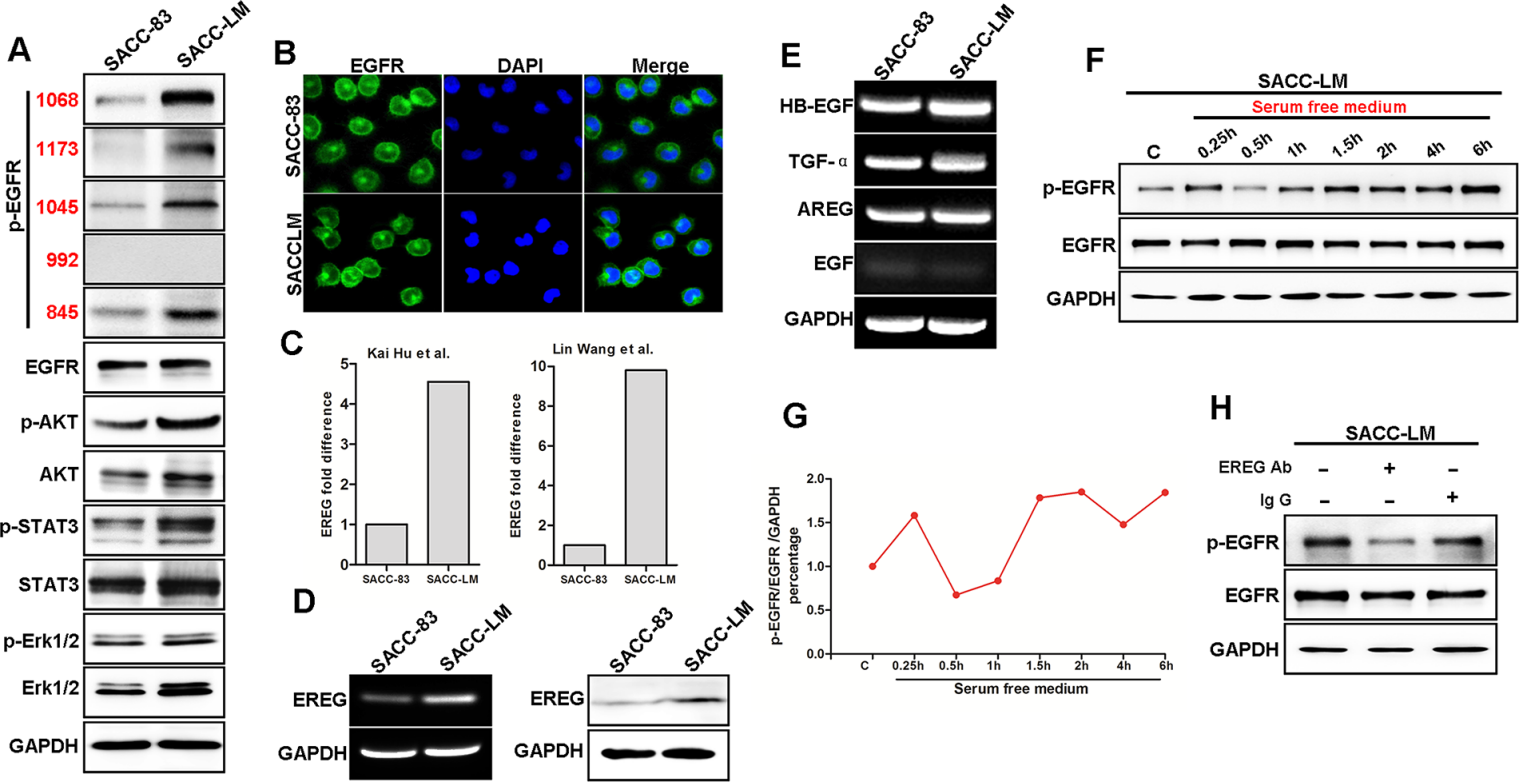
Autocrine EREG secretion contributes to the auto-activation of EGFR in highly metastatic SACC (**A**) Western blot analysis of p-EGFR, EGFR, p-AKT, AKT, p-STAT3, STAT3, p-ERK and ERK protein levels in SACC-83 and SACC-LM cell lines. (**B**) Immunofluorescence staining for EGFR is presented with DAPI (blue) nuclear staining. Scale bar = 200 μm. (**C**) Analysis of EREG mRNA levels with fold change in SACC-LM cells compared to SAC-83 cells using published chip assay data. (**D**) The mRNA and protein levels of EREG in SACC-83 and SACC-LM cell lines by RT-PCR and Western blot analysis, respectively. (**E**) The mRNA level of HB-EGF, TGF-α, AREG, EGF in SACC-83 and SACC-LM cell lines. (**F**) Western blot analyses of p-EGFR and EGFR from SACC-LM cells that were serum-starved as indicated. (**G**) Graphic representation of the ratio of p-EGFR and EGFR to GAPDH for indicated time points in SACC-LM cells. (**H**) Western blot analyses of p-EGFR in SACC-LM cells that were starved for 0.5h and then treated with EREG neutralizing antibody or with normal Ig G for 6 hours.

We assumed that differences in the signal transduction pathways of SACC subtypes were responsible for the lung-metastatic potential seen in SACC-LM cells. The EGFR is overexpressed in a variety of epithelial tumors, including salivary SACC. Activation of EGFR is thought to regulate the processes of metastasis and cancer cell survival. We examined phosphorylation of EGFR pathway target proteins in SACC-83 and SACC-LM cells. The results showed that p-EGFRs (Y1068, Y1173, Y1045, Y845) were all significantly increased in SACC-LM compared to SACC-83 (Figure 2A). Moreover, p-Akt, p-STAT3 and p-ERK were increased in SACC-LM compared to SACC-83 (Figure 2A). Of note, the EGFRs in SACC-LM were auto-activated since no exogenous ligand was added.

To determine if the EGFR in SACC-LM are mutated, we investigated genetic mutations by sequencing exons 18, 19, and 21 of the *EGFR* gene in both SACC-83 and SACC-LM cells; no genetic mutations were found in *EGFR* gene in either of these cell lines (data not shown). In addition, the subcellular localization of the EGFR showed no significant difference between the two cell lines (Figure 2B). Next, we asked if the differential activation of EGFR in these two SACC cell lines was the result of different levels of EGFR ligands. Previous reports of transcriptomic microarray analysis by Hu et al. [9], and by Wang et al. [33] showed that mRNA expression of EREG was 4.55-fold and 9.8-fold higher in SACC-LM than that in SACC-83, as determined by the respective investigators (Figure 2C). *EREG* encodes epiregulin, a known EGFR ligand. Thus, we examined mRNA and protein levels of EREG in these two cell lines by RT-PCR and immunoblot analysis. EREG mRNA expression was significant higher in SACC-LM than in SACC-83 (Figure 2D). Thus, autocrine secretion of EREG may contribute to an auto-activation of EGFR in SACC-LM cells. To determine if other EGFR-ligands were involved in EGFR activation, we examined the expression of EGF, TGFα, heparin binding-EGF (HB- EGF) and AREG in these two cell lines. However, there was no difference in the mRNA expression levels between these two cell lines (Figure 2E). To determine the role of autocrine cytokines, we examined protein expression after incubation in serum-free medium. The p-EGFR level was transiently decreased at 0.5 hour after the medium change, likely the result of removal of autocrine factor(s) in the conditioned (old) medium (Figure 2F–2G). Moreover, EGFR phosphorylation increased 1.5 hours after the medium change, suggesting that newly produced autocrine cytokines are responsible for this response (Figure 2F–2G). Importantly, a neutralizing anti-EREG antibody, but not normal Ig G, abrogated auto-phosphorylation of EGFR in SACC-LM cell (Figure 2H), which suggests that EGFR activation in SACC-LM cells is caused by the autocrine EREG. Thus, autocrine EREG likely contributes to the auto-activation of EGFR pathway in the highly metastatic SACC-LM cells.

### EREG is critical for activation of EGFR pathway and migration of SACC cells

**Figure 3:**
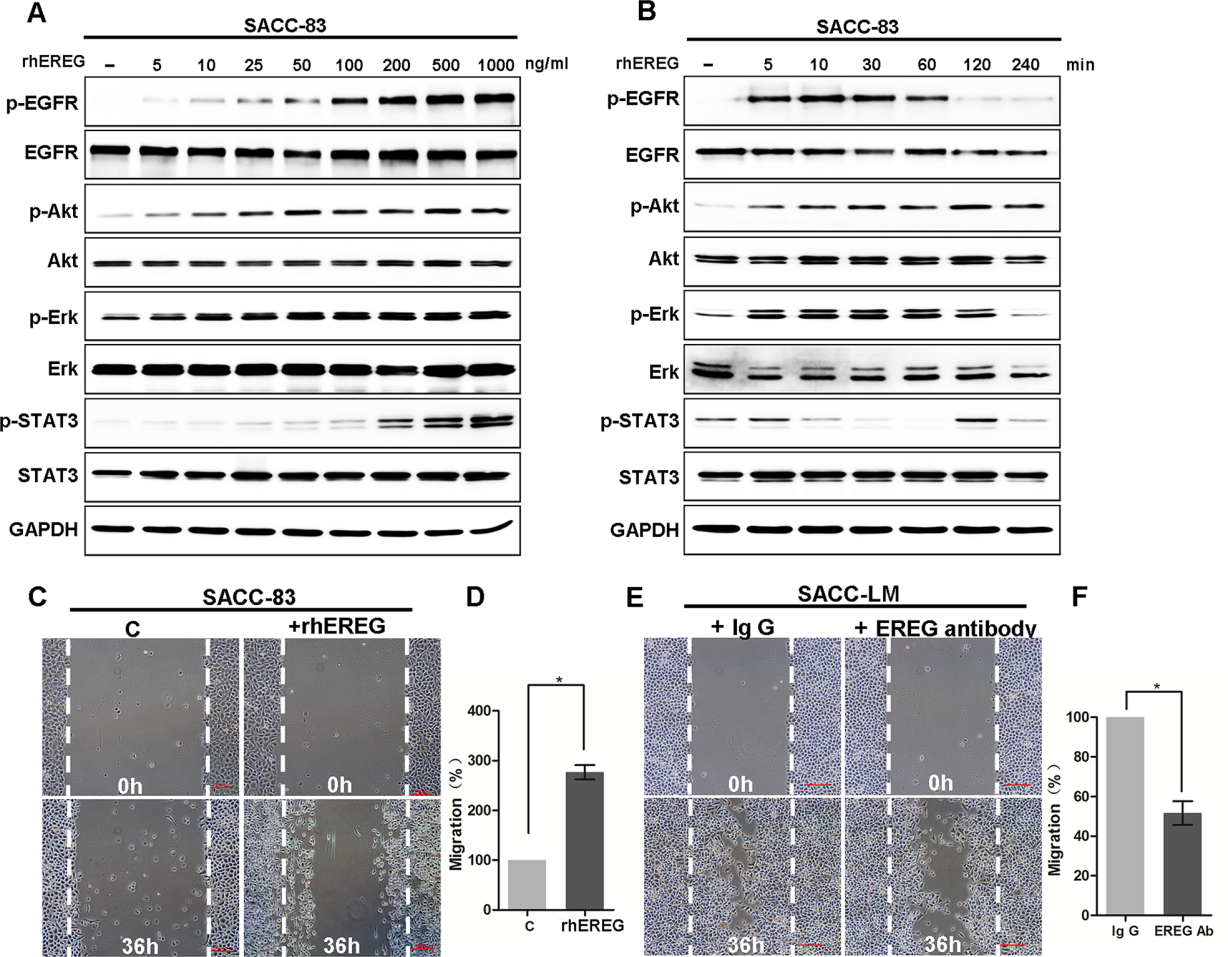
EREG activated EGFR/Akt/ERK/STAT3 pathways and promoted migration of SACC cells (**A**) Western blot analyses for P-EGFR, EGFR, P-AKT, AKT, P-ERK, ERK, p-STAT3, STAT3 in SACC-83 cells treated with 0 to 1000 ng/mL rhEREG for 4 hours. (**B**) Western blot analyses for P-EGFR, EGFR, P-AKT, AKT, P-ERK, ERK, p-STAT3, STAT3 in SACC-83 cells treated with 50 ng/mL rhEREG as indicated. (**C**) Representative images of migration by SACC-83 cells treated with or without rhEREG (50 ng/ml) for 36 hours with representative images shown. Scale bar = 200 μm. (**D**) Graphic representation of the percent of migrated cells treated with rhEREG from 3 separate experiments as outlined in (C) with the mean ± SD indicated, *p < 0.05. (**E**) Representative images of migration by SACC-LM cells treated with EREG neutralizing antibody or with normal Ig G for 36 hours with representative images shown. Scale bar = 200 μm. (**F**) Graphic representation of the percent of migrated cells treated with EREG antibody from 3 separate experiments as outlined in (E) with the mean ± SD indicated, *p < 0.05.

Next, we examined the effects of exogenous EREG on activation of EGFR and its downstream signaling targets, including ERK, Akt, and STAT3, in the SACC cell lines. Treatment of SACC-83 cells with recombinant human epiregulin (rhEREG) resulted in phosphorylation of EGFR, ERK, Akt and STAT3 in a time- and dose-dependent manner (Figure 3A–3B). Moreover, rhEREG treatment (50 ng/ml) significantly increased migration of SACC cells in the wound closure assay *in vitro* (Figure 3C). Importantly, a neutralizing anti-EREG antibody significantly inhibited wound closure whereas normal Ig G failed to do so (Figure 3F); this finding suggests that autocrine EREG is required for the migration activity of SACC-LM cells. Taken together, these results demonstrate that EREG plays a critical role in activation of the EGFR pathway and induction of SACC cell migration.

### EREG induces Snail and Slug protein stabilization

**Figure 4:**
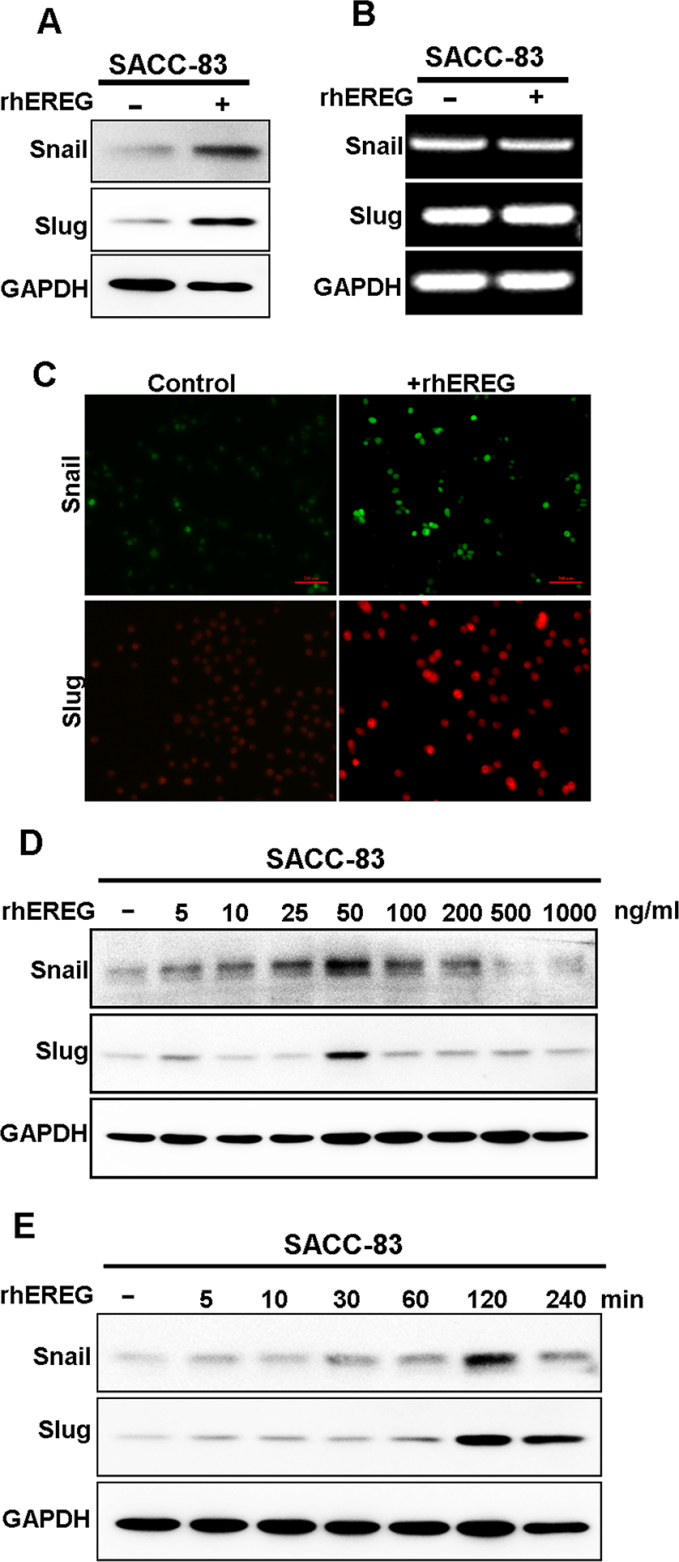
EREG induces protein stabilization of snail and slug (**A**) Western blot analyses for Snail and Slug in SACC-83 cells treated with or without 50 ng/mL rhEREG for 4 hours. (**B**) RT-PCR analyses for mRNA levels of Snail and Slug. (**C**) Immunofluorescence staining for Snail and Slug in SACC-83 cells treated with or without 50 ng/mL rhEREG for 4 hours. Scale bar = 200 μm. (**D**) Western blot analyses for Snail and Slug from SACC-83 cells treated with 50 ng/mL rhEREG as indicated. (**E**) Western blot analyses for Snail and Slug from SACC-83 cells treated with 0 to 1000 ng/mL rhEREG for 4 hours.

Snail and Slug are two major transcription factors that induce EMT and promote cancer metastasis. We next investigated whether Snail and/or Slug regulated EREG-induced SACC migration. Immunoblot analysis showed that rhEREG treatment significantly increased Snail and Slug protein levels in SACC-83 cells (Figure 4A), whereas mRNA levels of Snail and Slug, assessed by RT-PCR, remained unchanged (Figure 4B). Immunofluorescence microscopy confirmed these findings as the staining intensities of Snail and Slug were dramatically increased in SACC-83 cells following EREG treatment (Figure 4C). Thus, our results suggest that EREG regulated Snail and Slug expression via a transcription-independent mechanism. The optimal experimental condition for stabilization of Snail and Slug was rhEREG treatment for about 2 hour (Figure 4E) and at concentration of 50 ng/ml (Figure 4D).

### EGFR pathway is required for EREG-induced stabilization of Snail and Slug

**Figure 5:**
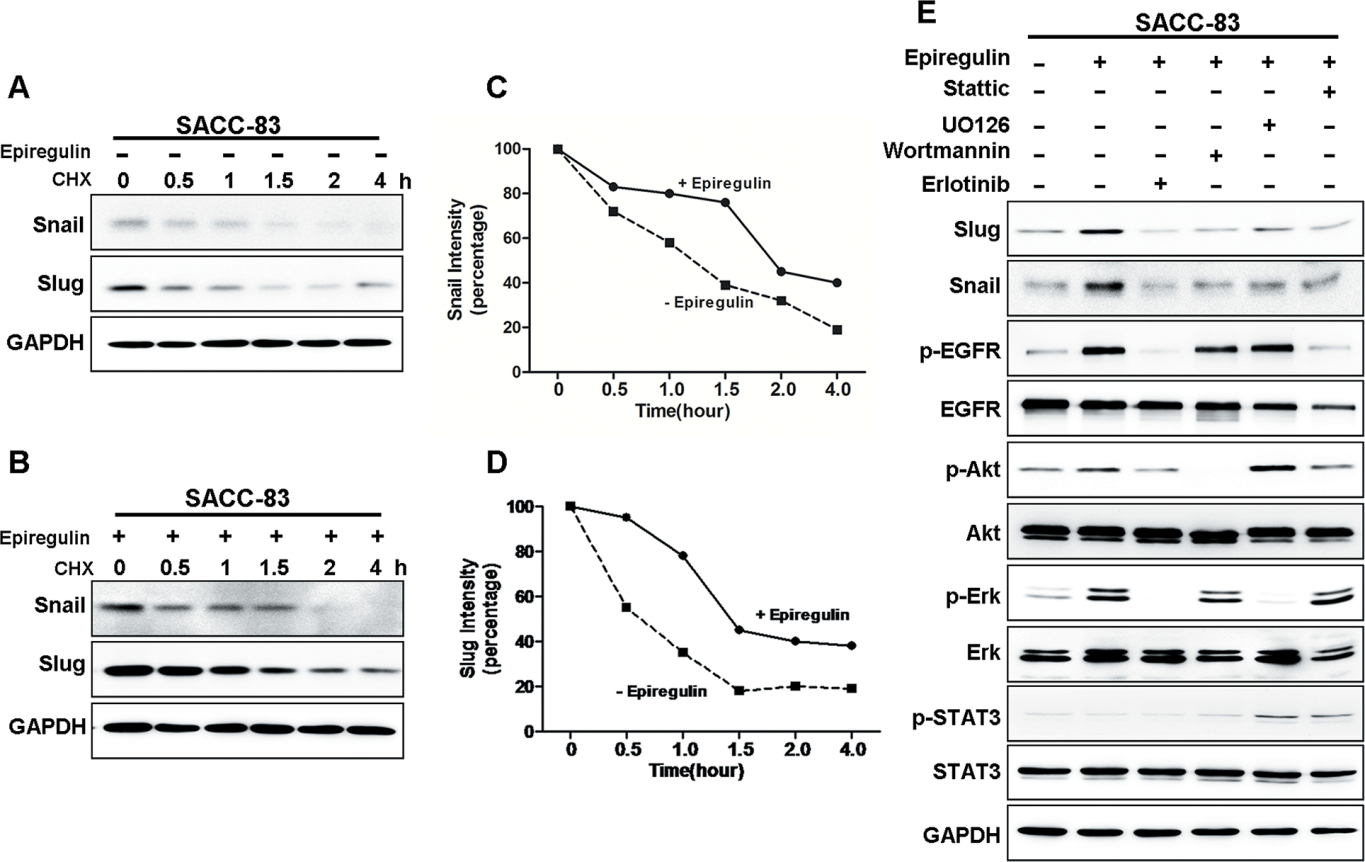
EREG enhanced Snail and Slug stability require EGFR activation (**A–B**) SACC-83 cells were treated with or without rhEREG for 2 hr, followed by incubation with cycloheximide (CHX; 10 μM) for an extended period of time. The levels of Snail and Slug were determined by Western blot analysis. (**C–D**) Graphic representation of densitometry results for Snail (C) and Slug (D) after cycloheximide treatment (circle, with rhEREG; square, without rhEREG). (**E**) Western blot analysis of Snail, Slug, P-EGFR, EGFR, P-AKT, AKT, P-ERK, ERK, p-STAT3, STAT3 from SACC-83 cells pretreated with various inhibitors for 1 hr followed by stimulation with rhEREG for 2 hr.

Snail and Slug are labile proteins [34]. To determine the mechanism underlying the EREG-induced stabilization, we examined the protein levels of Snail and Slug in cells treated with or without rhEREG for various time (up to 4 hour) in absence or presence of protein translational inhibitor cycloheximide (CHX) (Figure 5A–5B). In cells treated with rhEREG, Snail and Slug were more stable than cells without treatment (Figure 5C–5D). These results suggest that rhEREG treatment promotes stabilization of Snail and Slug. To determine which signaling pathway is essential for EREG-induced stabilization of Snail and Slug, we examined the effects of inhibitors of EGFR, Akt, STAT3, and ERK. The EGFR inhibitor erlotinib completely blocked EREG-induced stabilization of Snail and Slug, whereas inhibitors of STAT3, ERK and PI3K showed partial inhibition (Figure 5E). Because STAT3, MAPK and PI3K are the major downstream targets of EGFR, these results strongly suggest that the activated EGFR pathway is crucial for EREG-induced stabilization of Snail and Slug.

### Targeting EGFR suppressed migration and invasion of SACC-LM cells *in vitro*

**Figure 6:**
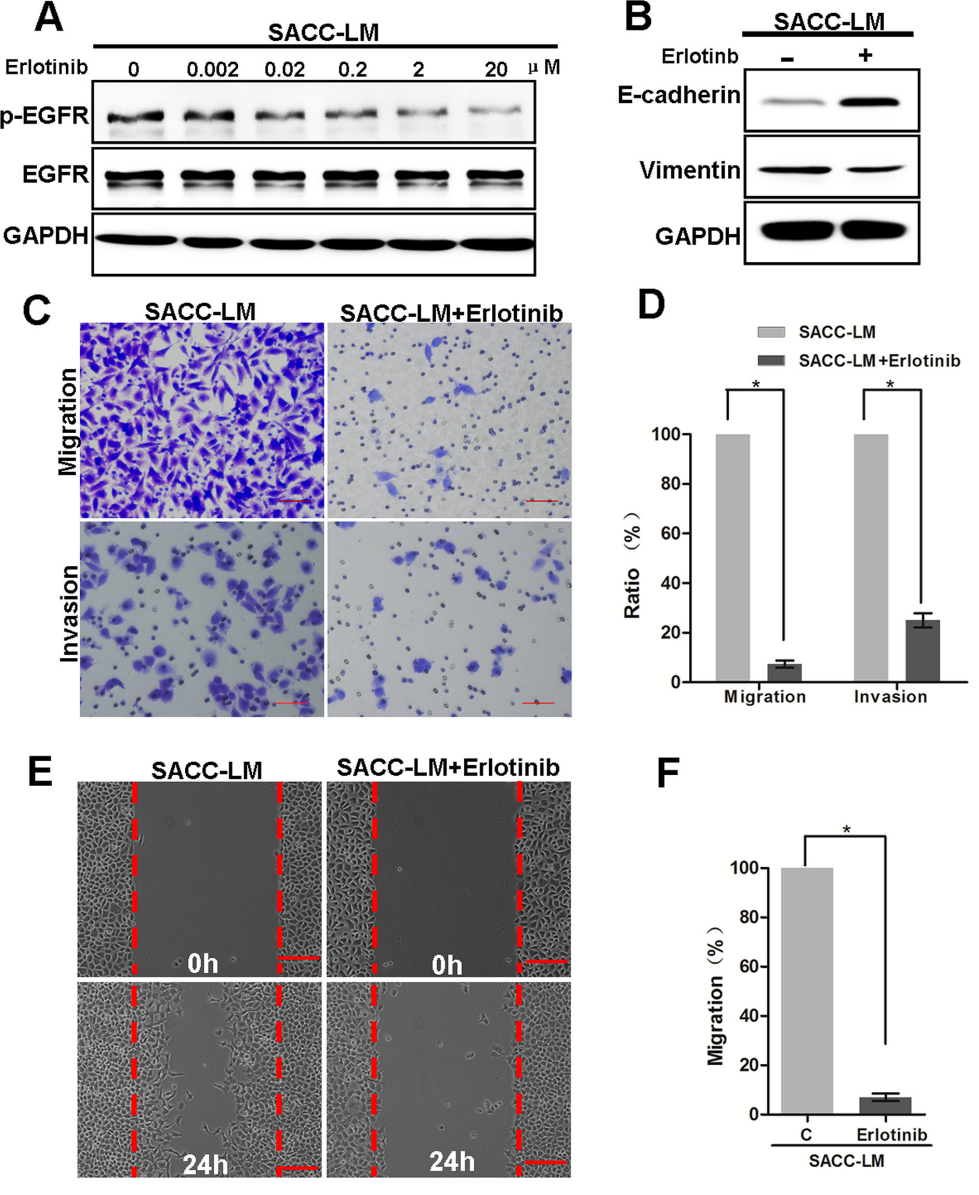
Targeting EGFR inhibits the migration and invasion capabilities of SACC-LM cells (**A**) Western blot analysis of p-EGFR and EGFR from SACC-LM cells treated with 0 to 20 uM erlotinib for 72 hours. (**B**) Western blot analysis of E-cadherin and vimentin levels from SACC-LM cells treated with 2 uM erlotinib for 72 hours. (**C**) Representative images of migration by SACC-LM cells treated with or without erlotinib (2 uM) for 24 hours. Scale bar = 200 μm. (**D**) Graphic representation of percent migrated cells from 3 separate experiments with mean ± SD percent indicated. * indicates a *p* < 0.05. (**E**) Representative images of wound healing by SACC-LM cells treated with or without erlotinib (2 uM) for 24 hours. Scale bar = 200 μm. (**F**) Graphic representation of the number of migrated cells within the areas of healing surpassing the red lines from 3 separate experiments. * indicates a *p* < 0.05.

Next, we examined the effects of targeting the EGFR on migration and invasion of SACC cells. Treatment of SACC-LM cells with erlotinib suppressed phosphorylated of the EGFR (Figure 6A), which correlated with decreased levels of vimentin and increased levels of E-cadherin (Figure 6B). Consistently, SACC-LM cell migration and invasion in the transwell assay were also dramatically suppressed by erlotinib treatment (Figure 6C–6D). Similarly, SACC-LM cell motility was also suppressed by erlotinib in wound healing assay (Figure 6E–6F). Taken together, these results demonstrate that the activated EGFR pathway is essential for the metastatic characteristics in SACC-LM cells, and that inhibition of EGFR function decreases the migration and invasion capabilities of these cells.

### Targeting EGFR suppressed lung metastasis of SACC-LM *in vivo*

**Figure 7:**
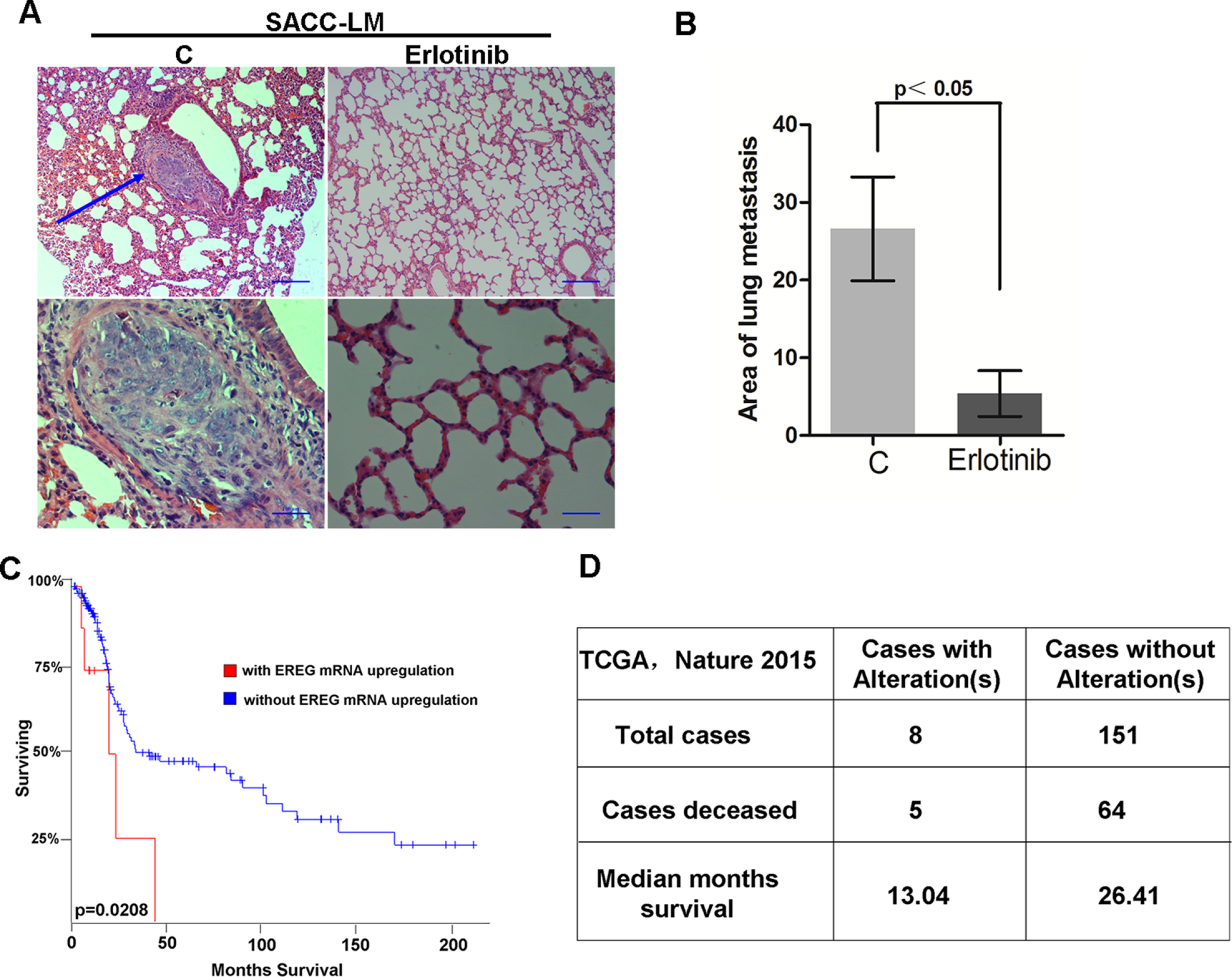
Targeting EGFR inhibits the lung metastasis of SACC *in vivo* (**A**) SACC-LM cells treated with or without erlotinib (2 uM) for 72 hours were injected into the tail vein of nude mice. Histopathologic analysis shows small metastatic nodules in lung tissues. (**B**) Graphic representation of the area of lung metastases with mean ± SD; *n* = 5. (**C–D**) Patient survival obtained from publicly available microarray data was analyzed based on EREG mRNA expression level.

Next, we examined the effect of targeted inhibition of the EGFR in SACC-LM cells on metastasis to the lungs *in vivo*. SACC-LM were pretreated without or with erlotinib at 2 uM for 72 hours, and then i.v. injected into nude mice. Four weeks later, mice were sacrificed. The metastatic nodules in mouse lungs were counted, and the total area of all of the nodules in each section of the lung was calculated. Erlotinib pretreatment greatly reduced both the number of metastatic nodules and the area of lung metastases when compared to control group (Figure 7A–7B). Thus, targeting EGFR significantly suppressed lung metastasis of SACC-LM *in vivo*.

To correlate the results from this study, we analyzed the clinical and molecular data of patients from the cancer genome atlas (TCGA). Because the survival data were not available for patients with SACC in the TCGA cohort, we selected one independent study on head and neck cancer (TCGA, Nature 2015). We performed analysis by using the cBio Cancer Genomics Portal (http://cbioportal.org) [35]. Specifically, the expression levels of EREG gene were divided into two groups, high expression and low expression groups. By analysis of the overall survival based on EREG mRNA expression, we found that patients with high EREG levels had a significantly shorter median overall survival than patients with low EREG levels (Figure 7C). The median survival for patients with EREG alterations was 13.04 months and 26.41 for patients without alterations (Figure 7D). Thus, the results obtained both in experimental and clinical data showed that the EREG-EGFR–Snail/Slug axis plays a critical role in controlling lung metastasis and in the prognosis of SACC patients.

## DISCUSSION

In this study, we showed that the highly metastatic SACC-LM cells exhibited the EMT-like characteristics compared to the low-metastatic SACC-83 cells. The EMT-like and metastatic features of SACC were promoted by autocrine EREG-activated EGFR signaling pathway. EREG-activated EGFR signaling stabilized Snail and Slug and induced EMT program in SACC-LM cells. Inhibition of EREG by neutralizing antibody abrogated EREG-induced EMT features in SACC-LM cells. Thus, autocrine EREG-induced EGFR activation, even without a genetic mutation, in metastatic SACC patients may be suitable for EGFR targeting therapy.

EGFR, a receptor tyrosine kinase, is important in controlling cell proliferation, motility, adhesion, invasion, angiogenesis and survival [36]. In fact, 85% of patients with SACC express the EGFR [37]. EREG is a ligand for the EGFR. Different from other members of the EGF family that are expressed ubiquitously in epithelium of normal tissues [38], EREG is predominantly expressed in the placenta and peripheral blood leucocytes. It has been reported that overexpression of EREG promotes migration and invasion of oral cancer cells *in vitro*. The effects of EREG are likely mediated by activation of ERK1/2, Akt, and Cox 2 [39]. Mechanistically, EREG activates not only homodimers of both ErbB1 and ErbB4 but also all possible heterodimeric ErbB complexes [23]. Thus, EREG may have important biological functions distinct from other EGFR ligands.

Distant metastasis and EMT program in SACC has also been associated with c-kit, another receptor tyrosine kinase [40]. c-kit was shown to increase the CD133^+^/CD44^+^ population in SACC [41]. And c-kit, along with Oct4, Nanog, SOX2, CD133, CD44, and ALDH1, has been considered as a cancer stem cell marker in primary non-small cell lung cancer (NSCLC), ovarian cancer and hepatocellular carcinoma [42]. Moreover, Slug was involved in the biologic activity of the SCF/c-kit signaling pathway [43, 44], and associated with an increased TNM stage, perineural invasion, local regional recurrence, and distant metastasis. However, targeting c-kit with inhibitors GISTs is not always effective in treating c-kit positive tumors [45]. This suggests that c-kit may not be the only or major molecule for mediating metastatic features, and other receptor tyrosine kinases may be involved in this metastatic process. Application of EGFR inhibitors, such as erlotinib and gefitinib, is a routine therapeutic targeting strategy for lung cancer patients with mutant EGFR, but not for those with wild-type EGFR. However, erlotinib appeared to be very effective in suppression of lung metastasis of SACC cells *in vivo*, as showed in this study. In support, some studies reported that gefitinib (Iressa), could stabilize 68% of SACC cases [46]. Although EGFR mutations in SACC [47, 48] are not as high as in lung cancer, the clinical effectiveness of targeting EGFR in SACC suggests that a different mechanism may be involved. Thus, targeting EGFR may be applicative for SACC patients with autocrine EREG, even no EGFR mutation.

In summary, we demonstrate that autocrine EREG-activated EGFR can stabilize Snail and Slug, induce EMT and metastatic features in SACC cells. Thus, the EREG-EGFR-Snail/Slug axis represents an important mechanism for SACC lung metastasis. Our study suggests that targeting EGFR may provide an effective therapeutic strategy for metastatic SACC with autocrine EREG and no genetic EGFR mutation.

## MATERIALS AND METHODS

### Cell cultures and reagents

Human SACC cell lines SACC-83 and SACC-LM were obtained from Shanghai 9th People's Hospital. Cells were cultured in RPMI-1640 (Gibco BRL, Grand Island, NY) with 10% fetal bovine serum (Gibco) and incubated in a humidified atmosphere of 95% air and 5% CO2 at 37°C. Recombinant Human epiregulin was obtained from R & D Systems (Minneapolis, MN, USA). Antibodies against GAPDH were from Santa Cruz Biotechnology (Santa Cruz, CA). Antibodies for E-cadherin, N-cadherin, vimentin, Snail, Slug, p-EGFR, p-AKT, p-ERK, p-SATAT3, EGFR, AKT, Erk and STAT3 were from Cell Signaling Technology Inc. (Beverley, MA,). Antibodies for ZO-1 and EREG and normal goat IgG were from Abcam (Cambridge, MA). HRP-conjugated secondary antibodies were from eBioScience (San Diego, CA).

### Western blotting

The experimental protocol was performed as described previously [49]. Cells were lysed with RIPA lysis buffer (Cell Signaling Technology). Cell lysates were separated by SDS-PAGE in a 10% acrylamide gel and transferred onto a nitrocellulose membrane for immunoblot analysis.

### Immunofluorescence

The experimental protocol was performed as described previously [50]. Cultured cells rinsed three times with PBS and fixed with 3.7% formaldehyde were permeabilized with 0.1% Triton X-100. After blocking in 1% BSA for 1 hour, cells were incubated with the primary antibody in a moist, 4°C chamber overnight, washed and then incubated for 1 hour with Alexa Fluor 488 (in the dark), or 594 donkey anti-rabbit IgG antibody (Invitrogen, Grand Island, NY) at room temperature. Washed cells (PBS containing 0.02% Tween 20), stained by mounting onto a slide with aqueous mounting medium containing 0.5 mg/ml 40–6-diamidino-2-phenylindole, were examined with a fluorescence microscope.

### Reverse transcriptase polymerase chain reaction (RT-PCR)

The experimental protocol was performed as described previously [51]. Total RNA samples were extracted with TriPure Isolation Reagent (Roche, Switzerland) and cDNA prepared from 1 mg of total RNA using the SuperScript III System (Invitrogen Life Technologies). The mRNAs levels were determined by RT-PCR, using the following primers:

EREG (F: 5′-ATCCTGGCATGTGCTAGGGT-3′ and R: 5′-GTGCTCCAGAGGTCAGCCAT -3′); HB-EGF (F:5′-CCACACCAAACAAGGAGGAG-3′and (F:5′-CCA GGAGGTCCGCATGCTCAC-3′and R:R:5′-ATGAGAA GCCCCACGATGAC -3′); TGF 5′-GAGTGCAGACCC GCCCGTGGC-3′); AREG (F:5′-AGGATCACAGCAGAC ATAAAG-3′and R: 5′-CCAAAACAAGACGGAAAGTG A-3′); EGF (F:5′-ATGTGTGCAGAGGGATACGC-3′and R: 5′-GGTGAGGAACAACCGCTACA-3′); GAPDH (F: 5′-TCCACCACCCTGTTGCTGTA -3′ and R: 5′-ACCAC AGTCCATGCCATCAC -3′); Snail (F: 5′-CGCGCTCTTT CCTCGTCAG -3′ and R: 5′-TCCCAGATGAGCATTGG CAG-3′); Slug (F: 5′- GAGCATTTGCAGACAGGTCA-3′ and R: 5′- CCTCATGTTTGTGCAGGAGA-3′).

### Transwell and wound healing assay

The experimental protocol was performed as described previously [52]. For the migration assay, cells (5 × 10^5^) were seeded onto the upper chamber in 200 μL of serum-free medium; the lower compartment was filled with 0.6 mL of DMEM media supplemented with 10% of FBS. After 24 h incubation, migrated cells on the lower surface of the filter were fixed and stained using 1% crystal violet; cells on the upper side were removed using a rubber scraper. Fluorescent images were obtained; reported data are counts of migrated cells with experiments performed in triplicate. The procedure used for invasion assay was similar to that of the cell migration assay, except that the transwell membrane was coated with Matrigel.

### Experimental lung metastasis model

This study was approved by the ethics committee of our institution. The experimental protocol was performed as described previously [22]. Cells (1 × 10^6^/0.2 ml) were injected into the tail vein of BALB/c nude mice. Animals were sacrificed after 5 weeks, and the metastatic tumors in the lung assessed. No mice showed notable toxic effects or body weight loss during the experiment. Visible lung metastatic nodules were counted macroscopically or in paraffin embedded sections stained with H & E. Data were analyzed using Student's *t* test; a *P* value less than 0.05 was considered significant.

### Statistical analysis

Data analysis used SPSS (Statistic Package for Social Sciences) 13.0 for Windows (SPSS Inc., Chicago, IL, USA). Unpaired Student's *t*-tests or U-Mann Whitney tests determined statistical significance between groups with *P* values < 0.05 considered significant.
